# Liquid-liquid phase separation in DNA double-strand break repair

**DOI:** 10.20892/j.issn.2095-3941.2023.0252

**Published:** 2023-09-19

**Authors:** Jia Chen, Jie Shi, Jian Zheng, Yunlong Wang, Xiangbo Wan

**Affiliations:** 1 Henan Provincial Key Laboratory of Radiation Medicine, The First Affiliated Hospital of Zhengzhou University, Zhengzhou 450052, China; 2 Academy of Medical Sciences, Zhengzhou University, Zhengzhou 450052, China; 3 Department of Radiation Oncology, The First Affiliated Hospital of Zhengzhou University, Zhengzhou 450052, China; 4 Department of Radiation Oncology, The Sixth Affiliated Hospital, Sun Yat-sen University, Guangzhou 510655, China; 5 Guangdong Provincial Key Laboratory of Colorectal and Pelvic Floor Diseases, The Sixth Affiliated Hospital, Sun Yat-sen University, Guangzhou 510655, China

DNA repair factors function through spatiotemporal condensation and dissolution at DNA double-strand breaks (DSBs). Recent advances have indicated that some DSB repair factors undergo liquid-liquid phase separation (LLPS) and show droplet-like properties, as well as dynamic material exchange. Importantly, LLPS regulates various biological processes, and aberrant LLPS is involved in the pathologic progression of various diseases.

In addition, the phenotype of dynamic condensation and dissolution of DNA repair factors during DSB repair presents properties to LLPS. Significantly, RNA, poly (ADP-ribose) [PAR], and post-transcription modification (PTM), such as phosphorylation, ubiquitination, and methylation, are thought to contribute to the LLPS of DSB repair factors. From the perspective of LLPS function during DSB, DNA repair factors may undergo LLPS to act in DSB sensing and DNA damage repair signal transduction, participate in homologous recombination (HR)- and non-homologous end-joining (NHEJ)-mediated DSB repair, and regulate downstream responding pathways. Based on these findings, researchers have focused on exploring the DNA repair factors with LLPS characteristics and clarifying the functions.

## Brief introduction on DSBs and DSB repair

DSBs, the most toxic type of DNA damage to cells, are caused by replication stress, chemotherapy, radiotherapy, or ionizing radiation (IR)^1,2^. In general, NHEJ, HR, and alternative end-joining (Alt-EJ) are major DSB repair pathways. After DSBs occur, the phase of the cell cycle and the properties of DSB ends are identified as vital elements for repair pathway choice. The dominant NHEJ is error-prone and functions through the entire cell cycle, while HR is error-free and mainly activated in the S and G2 phases; Alt-EJ occurs in NHEJ-deficient cells^2^.

**Figure 1:**
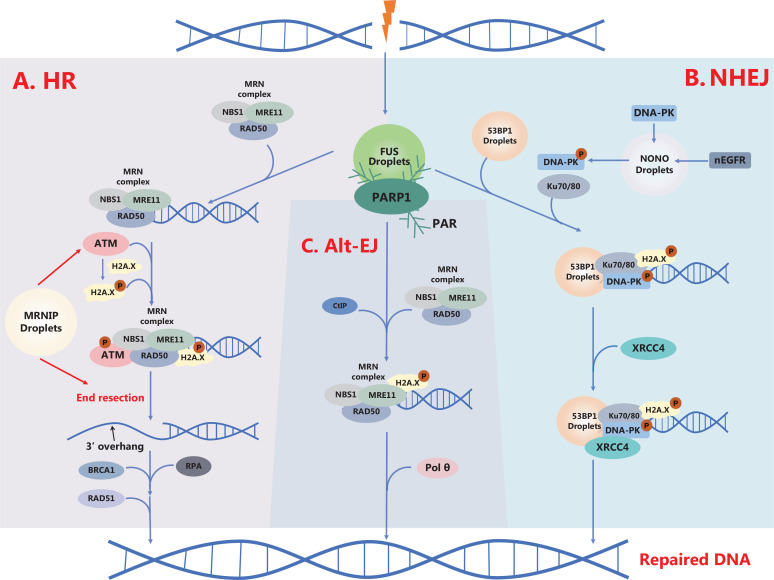
The roles of LLPS in DSB repair pathways. (A) The HR pathway is an error-free repair pathway and MRNIP LLPS promotes DSB sensing and end resection. (B) The NHEJ pathway is an error-prone pathway. NONO LLPS enhances DSB repair by accelerating nEGFR-induced DNA-PK phosphorylation; 53BP1 LLPS is required for its role in NHEJ. (C) The alternative end joining (Alt-EJ) pathway is an alternative NHEJ pathway.

DNA damage response (DDR) is triggered by DSBs and are mainly divided into three steps. First, DNA damage is recognized by DNA damage sensor proteins that are recruited to DSB sites in seconds, such as the MRE11-RAD50-NBS1 (MRN) complex and Ku proteins (Ku70/80), during the sensing process^2^. Second, DNA damage sensor proteins located at DSBs recruit downstream signal transducers, such as ATM, ATM and Rad3 related (ATR), and checkpoint kinase 1 (CHK1), which amplify DNA repair signals and promotes the DDR in the initiating cascade reaction stage^1^. Interestingly, DDR-related gene mutations have been reported to correlate with clinical outcomes. For example, Wang et al.^3^ reported that in gastrointestinal (GI) cancer, the presence of > 2 DDR gene mutations is associated with prolonged overall survival (OS) and a higher tumor mutation burden, which may provide guidance in GI clinical immunotherapy^3^. Third, the damaged DNA is repaired by the HR, NHEJ, or Alt-EJ pathways during the repair stage. During HR, the DSB ends are processed to generate a 3′-single-stranded DNA (3′-ssDNA) overhang, which assembles with RAD51 and BRCA1 to form nucleic acid-protein complexes for the subsequent repair process^2^ (**Figure 1A**). As a principal pathway of DSB repair, NHEJ drives the direct joining of DNA ends by multiple protein-synaptic complexes^2^ (**Figure 1B**). Alt-EJ is an effective way to connect resected DNA ends directly *via* DNA polymerase theta (Pol θ), which depends on the micro-homologous sequences near the ends of DSBs^1^ (**Figure 1C**).

## Brief introduction of LLPS

The phenomenon of LLPS was first discovered in P granules of *Caenorhabditis elegans*, which is described as a liquid-like organelle^4^. Typically, organelles of eukaryotic cells include membrane-compartmentalized and membrane-less organelles. Using imaging-based techniques, including fluorescence recovery after photobleaching (FRAP) and fluorescence loss in photobleaching (FLIP), emerging advances indicate that the formation of membrane-less organelles may be driven by phase separation, and exhibit liquid-like properties^5^.

**Figure 2:**
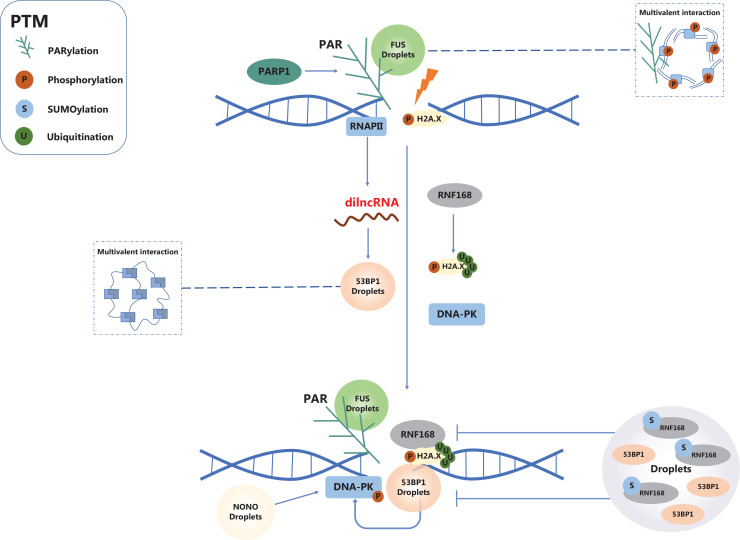
The regulation of DSB repair-related LLPS by RNAs, PARylation, and PTMs. dilncRNAs, PARylation, and SUMOylation regulate the LLPS of DNA repair factors.

The primary driving force of LLPS is multivalent interactions between biomolecules, which are described as two main forms: intrinsically disordered regions (IDRs) mediate weak multi-valent interactions among the same protein molecules (**Figure 2**); and modular interactions mediate multi-valent interactions among the different molecules. IDRs are also designated as low-complexity domains (LCDs)^6^. For example, an IDR at the N-terminal of fused in sarcoma (FUS) protein is required for FUS phase separation^7,8^ (**Figure 2**). The interaction between modular domains, especially proteins containing multiple modular interactions, also provides strong multivalent interactions to trigger LLPS. For example, multivalent interactions among nephrin, a transmembrane protein expressed by kidney podocytes, neural Wiskott-Aldrich Syndrome protein (N-WASP), and Nck (binding proteins of the WASP family) have been discovered to assemble LLPS^6^.

It has been shown that LLPS participates in diverse bio-physiologic processes, including transcription activation and depression, and chromatin organization^5,6^. Consistent with a pivotal role in cellular activities, dysregulation of LLPS has been identified as the mechanism underlying many diseases, including cancer and immunologic disorders^6^. Specifically, aberrant phase separation may result in protein aggregation, leading to the development of neurodegenerative diseases (NDs), such as Alzheimer’s disease (AD) and amyotrophic lateral sclerosis (ALS)^9^.

## LLPS functions in DSB repair

Generally, three main features are used to differentiate LLPS puncta from classic DNA repair foci. First, the FRAP assay shows that protein has a rapid exchanging dynamic between LLPS puncta and surroundings. Second, liquid-like properties, such as fusion between adjacent puncta or fission of a big punctum to smaller puncta, are observed in live cell imaging. Finally, the purified protein undergoes LLPS *in vitro*. Many DNA repair factors have been identified that exhibit LLPS properties, which are essential for the DSB repair process. These LLPS factors participate in every stage of the DNA damage response, including DSB sensing, signal transduction and amplification, repair processes, and downstream responding signaling^7,8,10,11^.

### LLPS in DSB sensing and signal transduction

When DNA is damaged, several DSB LLPSs are required to sense the damage, and trigger DDR factors to form the DNA repair center complexes, then initiate DDR. To be specific, PAR triggers the aggregation of PAR-associated IDR-containing proteins at DSB sites, such as FUS, Ewing sarcoma (EWS), and TATA box-binding protein-associated factors (TAF15). The interaction between PAR and FUS is thought to be a vital mechanism underlying the DSB sensing process. Specifically, in response to DNA damage, PARP1 is activated and catalyzes the synthesis of long PAR chains, which bind to FUS to stimulate FUS LLPS^11^ (**Figure 2**). FUS-dependent LLPS is necessary for initiating DDR and is required at an early stage for the stability of DSBs, including recruiting DSB sensor proteins to damaged DNA, such as Ku70/80. In addition, recruitment of splicing factor proline- and glutamine-rich (SFPQ) and relocation of 53BP1 to DNA damage sites is promoted by FUS-induced LLPS^7^. Furthermore, known as a critical DNA repair factor in HR, Oshidari et al.^12^ reported that Rad52 shows liquid properties in yeast, which coordinates with various kinds of DNA damage-inducible intranuclear microtubule filaments (DIMs) and serves as a guide to assemble DNA repair center droplets^12^. In addition, FUS LLPS sustains the integrity of DNA damage foci and is required for γH2A.X cluster formation, therefore further mediating DDR signaling cascade activation^7^.

LLPS also participates in the interaction of DSB repair sensors with signaling transducers. Our group found that MRN-interacting protein (MRNIP) forms liquid-like condensates that recruit and concentrate the MRN complex. After a DSB occurrence, MRNIP droplets rapidly moves to the damaged DNA to accelerate the sensing of DSB by the MRN complex, resulting in enhanced ATM activation^8^ (**Figure 1A**). Interestingly, in this study we performed a screen to identify DNA repair factors containing large IDRs and found that approximately 10% (56/533) of DNA repair-related genes were largely disordered regions with a predictor of natural disordered regions (PONDR) score > 0.7, suggesting that this may be a common feature for DNA repair factors^8^. Recently, a study showed that SUMOylated RNF168 undergoes phase separation to reduce accessibility to DSBs, restricting the recruitment of RNF168 to DNA damaged sites^13^ (**Figure 2**).

### LLPS in the DSB repair process

In NHEJ-mediated DNA repair, 53BP1 participates in DSB repair by facilitating NHEJ and restraining HR. Pessina et al.^9^ first characterized 53BP1 LLPS and reported that 53BP1 LLPS is essential for DSB repair in cells. Non-POU domain-containing octamer-binding (NONO) is a DDR-related RNA-binding proteins (RBPs) that is associated with the DSB repair pathway choice. Our studies demonstrated that NONO undergoes LLPS to form liquid-like condensates to recruit nuclear EGFR and DNA-PK, enhancing DSB-induced DNA-PK activation to promote NHEJ-mediated DNA repair^14^ (**Figure 2**). As another core RBP in DSB repair, SFPQ forms phase separation with the help of FUS and is induced by paraspeckles of lncRNA. Similar to NONO, SFPQ LLPS may implicate DNA repair and promote NHEJ^7^.

The MRN complex-mediated DNA end resection is pivotal for HR-mediated DSB repair. Our study showed that MRNIP LLPS enhanced loading of the MRNIP complex to the DSB end, thus accelerating the DNA end resection and HR-mediated DSB repair. Most importantly, MRNIP LLPS was correlated with the response to tumor radiotherapy, suggesting that MRNIP LLPS may be used as a biomarker for the radiotherapy response^8^. Interestingly, when DSBs occur, plant-specific histone methyltransferase SUVR2 (MtSUVR2) has been shown to maintain genome integrity by LLPS. MtSUVR2 undergoes LLPS to form condensates induced by DNA damage and located at DSB sites. MtSUVR2 LLPS in the nucleus is dependent on the IDR1 and LCD domains. In addition, MtRAD51 LLPS is driven by MtSUVR2 and has been discovered to participate in HR-mediated DNA repair^15^.

### LLPS in DSB-mediated p53 activation

Tumor suppressor protein p53 has a central role in DSB-related cell cycle regulation. Specifically, 53BP1 LLPS serves as a scaffold for p53 and p53-activating proteins and provides a suitable environment to permit p53 activation, which suggests that 53BP1 transmits the signal from damaged DNA to global activation of p53 and coordinates cell cycle checkpoint activation^10^. Another DSB repair factor that has been reported to regulate p53 is the nucleolar protein, NOP53. Shi et al.^16^ reported that NOP53 is located in nucleoli and undergoes LLPS driven by its IDR, which negatively regulates irradiation-induced p53 activation. High expression of NOP53 is correlated with a poor prognosis in CRC patients receiving radio-chemotherapy^16^.

## Mechanism underlying DSB repair factors undergoing LLPS at DSB sites

### RNA

Damage-induced long non-coding RNA (dilncRNA) has been shown to drive 53BP1 protein LLPS at DSB sites. When a DSB occurs, RNA polymerase II binds to the MRN complex and is recruited to DSB sites, then induces formation of dilncRNA. Promoted by dilncRNA, 53BP1 is condensed as DDR foci and shows LLPS properties at DSB sites, such as dynamic property and liquid-like behavior. Moreover, anchored at damaged DNA, dilncRNA forms DNA-RNA hybrids and enhances HR with the 53BP1 interaction^17^. In addition, 53BP1 has the capacity to interact with methylation of histone H4 lysine 20 (H4K20me2). Therefore, interactions among 53BP1, dilncRNA, and PTMs, which facilitate the subsequent DSB repair process, such as HR-mediated DNA repair^9^.

### Poly(ADP-ribosyl)ation [PARylation]

PARylation is considered an essential and reversible PTM during the DNA repair process. A high PARylation level of RBPs is assumed to function in DDR. As a highly PARylation nuclear RBP, FUS interacts with PAR chains that are synthesized by PARP1 (**Figure 2**). PARylation FUS undergoes LLPS at DSB sites and forms damaged DNA-rich compartments, then triggers rapid DNA damage repair^11,18^.

### PTM

PTMs, such as phosphorylation, ubiquitination, and methylation of chromatin, interact with the C-terminus of 53BP1. To be specific, the C-terminus of 53BP1 contains the ubiquitination (Ub)-dependent recruitment (UDR) and two tandem Tudor domain, which binds to ubiquitinated H2AK15 and H4K20me2. Therefore, chromatin modification, such as phosphorylation, ubiquitination, and methylation, may support 53BP1 undergoing LLPS at damaged DNA^1,9^. Additionally, aberrant PTMs of LLPS factors are also related to several pathologic diseases. For example, tau phosphorylation facilitates phase separation *in vitro* and acts as a hallmark of AD^19^.

## Conclusions and perspectives

As previously described, LLPS has important roles in the DSB sensing process, the initiating cascade reaction stage, and the DNA repair pathways of NHEJ and HR. However, the Alt-EJ, which is considered an alternative to NHEJ and HR in the G2 phase, still remains to be further explored and the roles of LLPS in the Alt-EJ pathway are not clear^1^. In the context of DSB repair, although lots of breakthroughs of LLPS have been obtained, there are still some deep questions that need to be further elucidated. First, the subsequent recruitment and dissolution of a variety of DSB repair factors at the DSB site are required for the completion of DSB repair, while all studies involving DSB repair factor LLPS have focused on a single protein, in which LLPS properties were characterized and function was elucidated. How do DSB factors undergo LLPS in an accurate sequence at the DSB end? Is there interplay among different DSB factor LLPS? What is the underlying mechanism? In addition, the LLPS properties of chromatin have been identified for nearly a decade. Is there any interplay between the DSB repair factor LLPS and chromatin? Answers to all these questions would deepen our understanding of the DSB repair mechanism.
